# PRIOR TECHNOLOGY USE AND INTERVENTION OUTCOMES: INSIGHTS FROM A HEARING CARE TRIAL WITH AMPLIFICATION DEVICES

**DOI:** 10.1093/geroni/igae098.0432

**Published:** 2024-12-31

**Authors:** Jonathan Suen, Ethan Wang, Joshua Betz, Emmanuel Garcia Morales, Jami Trumbo, Nicole Marrone, Hae-Ra Han, Carrie Nieman

**Affiliations:** Johns Hopkins University, Baltimore, Maryland, United States; Johns Hopkins University, Baltimore, Maryland, United States; Cochlear Center for Hearing & Public Health, Baltimore, Maryland, United States; Cochlear Center for Hearing & Public Health, Baltimore, Maryland, United States; Johns Hopkins University, Baltimore, Maryland, United States; University of Arizona, Tucson, Arizona, United States; Johns Hopkins University, Baltimore, Maryland, United States; Johns Hopkins University School of Medicine, Baltimore, Maryland, United States

## Abstract

The total number of older adults in the U.S. with clinically significant hearing loss is projected to grow by an estimated 70% by the year 2060. Nevertheless, the adoption of hearing care strategies such as amplification technologies remains low nationally. Evidence of population disparities in accessing respective care along with adverse health outcomes linked to untreated hearing loss are also increasing, warranting a public health response. The Hearing health Equity through Accessible Research and Solutions (HEARS) intervention offers a novel care model leveraging OTC technologies for community-dwelling older adults with hearing loss and low income. A secondary analysis of data from a community-based RCT investigated the effect modification of self-reported prior use of technologies such as smartphones and computers on intervention outcomes across 151 participants. Participants classified as “tech-connected” showed a -14.1 point decrease (95% CI: -17.6, -9.9) on a standardized measure of hearing-related communication impairments when compared to a control group while “not tech-connected” participants demonstrated a -12.5 point decrease (95% CI: -16.2, -5.2), with a greater decrease indicating more favorable outcomes. The estimated difference in treatment effects of -1.55 points (95% CI: -7.8, 4.1) however was not statistically significant, revealing that prior technology use did not modify intervention outcomes among the RCT’s cohort. Nonetheless, technology literacy remains an important factor for hearing care intervention research to consider when designing outreach to underserved older populations. Key considerations related to prior technology use will be outlined and described for programs reliant upon OTC amplification technologies in addressing age-related hearing loss.

